# Association between GDF15, poverty and mortality in urban middle-aged African American and white adults

**DOI:** 10.1371/journal.pone.0237059

**Published:** 2020-08-07

**Authors:** David W. Freeman, Nicole Noren Hooten, Yoonseo Kim, Nicolle A. Mode, Ngozi Ejiogu, Alan B. Zonderman, Michele K. Evans

**Affiliations:** Laboratory of Epidemiology and Population Science, National Institute on Aging Intramural Research Program (NIA IRP), National Institutes of Health (NIH), Baltimore, Maryland, United States of America; University of Central Florida, UNITED STATES

## Abstract

Mortality disparities are influenced by race and poverty. There is limited information about whether poverty influences biologic markers of mortality risk. Emerging data suggests that growth differentiation factor 15 (GDF15) is associated with mortality; however, the interplay between GDF15, sociodemographic factors and mortality is not known. We sought to evaluate the interactions between GDF15 and sex, race and poverty status on mortality. Serum GDF15 was measured in 1036 African American and white middle-aged men and women above and below 125% of the Federal poverty status from the Healthy Aging in Neighborhoods of Diversity across the Life Span (HANDLS) study. Multivariable adjusted Cox regression models were used to assess the association between log-transformed GDF15 (logGDF15) and 12-year mortality outcomes (all-cause, cardiovascular- and cancer-specific outcomes) and interactions with sex, race and poverty status. Likelihood ratio tests were used to assess significance of the interaction terms. Median GDF15 was 655.2 pg/mL (IQR = 575.1). During 12.2 years of follow-up, 331 died of which 94 cardiovascular- and 87 were cancer-specific deaths. One unit of increase in logGDF15 was associated with a hazard ratio for all-cause mortality, cardiovascular- and cancer-specific mortality of 2.26 (95% confidence interval [CI], 1.94–2.64), 2.74 (95%CI, 2.06–3.63) and 1.41 (95%CI, 1.00–2.00), respectively. There was an interaction between logGDF15 and poverty status on all-cause mortality (p<0.05). The GDF15×poverty status interaction term improved model calibration for all-cause mortality. Our study provides the first evidence that the effect of elevated GDF15 on all-cause mortality is modified by poverty status.

## Background

Although overall mortality rates continue to decline in the United States, recent trends document disproportionate increases in mortality and life expectancy amongst groups in the United States [1]. Notably, age-adjusted death rates for non-Hispanic African American men increased [2]. Additionally, there were increases in the age-specific death rate for all Americans younger than 65 years of age; this is particularly true for midlife whites between the ages of 25–64 [3]. Low socioeconomic status remains a significant risk factor for morbidity and premature mortality [4]. Specifically, African Americans (AAs) below poverty are particularly vulnerable to early death compared to their white counterparts despite adjustments for various lifestyle cofactors [5, 6]. Disentangling the effects of race and poverty on mortality and other negative health related outcomes are difficult given the low median household income and high poverty rate among AAs [7]. Given this disparity, it is important and useful to evaluate relevant biological markers of mortality in the context of populations at risk for premature mortality in hopes of improving health outcomes for these individuals.

Mortality is linked to numerous behavioral or physiologic risk factors including tobacco use, alcohol consumption, obesity, physical inactivity, elevated blood pressure and hyperlipidemia [8]. In addition, other biological and clinical measures have been associated with or predict risk of mortality including interleukin-6 [9], C-reactive protein [9], albumin, alkaline phosphatase, estimated glomerular filtration rate (eGFR), blood urea nitrogen (BUN), mean cell volume (MCV) [10] and red cell distribution width (RDW) [11]. Several groups have shown recently that growth differentiation factor 15 (GDF15), also known as MIC-1, is an independent predictor of all-cause mortality in white elderly (>70 years) individuals [12–15], whites with coronary heart disease [16] and also in white middle-aged individuals [17]. Data from the Dallas Heart Study suggest that GDF15 may also be associated with mortality in a multiethnic cohort [18]. Circulating GDF15 levels are also a biomarker of cardiovascular disease (CVD) and its progression [19].

GDF15 is a multifunctional secreted protein expressed at low levels in most tissues and upregulated in response to tissue stress or injury [20]. GDF15 is an emerging biomarker and a stress response cytokine produced in response to stressors, cellular and systemic injury and inflammation. Data suggest that it is a pleiotropic signaling molecule that has both beneficial or advantageous effects on cellular metabolism but may in certain biologic contexts have deleterious consequences as well [21]. Under physiological conditions, GDF15 is produced in small amounts by the liver, lung and kidney but may be expressed in large amounts following exposure to external and internal stressors [20]. Its production is regulated by various stress responses, senescence and inflammation-related genes such as *CRP*, which regulates GDF15 in a p53 dependent manner [22], and *IRE1alpha* following endoplasmic reticulum stress [23]. However, these actions of GDF15, which are transduced through the glial-cell-derived neurotrophic factor (GDNF) receptor α-like (GFRAL) receptor and the tyrosine kinase coreceptor RET [24–27], are time and context dependent i.e. organ and system specific. Accumulating evidence, largely from animal models, suggests that during early stages of chronic inflammation, cancer and metabolic disorders, the effects of GDF15 are aimed at limiting disease process. However, in advanced cancer, heart failure or renal disease, GDF15 causes the anorexia-cachexia syndrome and also may promote metastases [20]. Furthermore, recent work indicates that GDF15 plays an important role in energy homeostasis and body-weight regulation [24–27].

Most previous studies on the association between GDF15 and mortality risk (overall and cause-specific) were conducted in highly selected population groups such as randomized controlled trials, hospitalized patients, elderly individuals and critically ill patients who are not representative of the general population. Apart from the study by Rohatgi *et al*.[18], investigations of GDF15 and mortality outcomes in population-based studies were conducted in individuals of European-ancestry with socioeconomically homogeneous individuals. Hence the role of GDF15 on the risk of mortality in racially and socioeconomically diverse populations who are at-risk of health disparities and premature mortality has been unclear. At present, it is not known whether GDF15 interacts with any socioeconomic and demographic factors or other biomarkers of mortality. Given the persistence of health disparities and disproportionate burden of disease and mortality borne by AAs evidenced by the ongoing Covid-19 pandemic [28, 29], it is critical that we examine the utility of mortality and disease biomarkers to identify populations at heightened risk. It is particularly important to examine the potential influence or interaction of poverty, a dominant social determinant of health that drives differential health outcomes.

The objectives of the present study were to assess the association between serum GDF15 and all-cause and cause-specific mortality, and to identify interactions between GDF15 and sex, race and poverty status in a large cohort of community-based middle-aged adults recruited from Baltimore, Maryland.

## Methods

### Study population

The Healthy Aging in Neighborhoods of Diversity across the Life Span (HANDLS) study of the National Institute on Aging Intramural Research Program, National Institutes of Health (NIH) is a prospective, community-based longitudinal cohort study based in Baltimore, Maryland. Participants were recruited based on a factorial cross design of sex, race, 5-year age group and poverty status, defined as above or below the 125% of the 2004 US federal poverty guidelines (https://handls.nih.gov/) [30]. A total of 3720 self-identified African American and white participants aged 30–64 who resided in Baltimore, MD were recruited at baseline. HANDLS has been approved by the Institutional Review Board of the National Institute of Environmental Health Sciences, NIH. All participants provided written informed consent.

### Mortality follow-up

Mortality was ascertained by matching with the National Death Index (NDI), National Center for Health Statistics, for all participants from enrollment (2004–2009) through December 31, 2016. NDI data included date and primary cause of death (International Classification of Diseases 10^th^ edition). There were 331 deaths (all-causes) after 12.2 years follow-up (median 9.2 years). CVD-specific death was defined as ICD10 codes I00–I99 and cancer-specific death (malignant neoplasms) was defined as C00–C96 (www.icd10data.com). Power was calculated using the ssizeEpi.default function in the *powerSurvEpi* R package. Sample size calculation revealed, after accounting for five percent assay failure rate, a total of 1037 samples (706 controls and 331 deaths) were needed to detect a hazard ratio of at least 1.75 for all-cause mortality associated with elevated GDF15 at alpha value of 0.05 and a power of 80%. The effect size to estimate the sample size was taken from Hagstrom et al. [16].

### Measurement of serum GDF15 protein levels

Blood samples were collected at study enrollment (2004–2009) in vials with no additives, centrifuged and serum was aliquoted and immediately frozen at -80°C until use. Serum GDF15 (pg/mL) were quantified using the Quantikine ELISA kit (R&D Systems, Cat#SGD150, Minneapolis, MN) according to the manufacturer’s instructions. Serum was diluted 1:4 in calibrator diluent RD5-20 and 50 μl was used for the assay. Three inter-assay and within assay controls consisting of serum pooled from 6 individuals were present on each plate and an internal standard on each plate was used to calculate GDF15 concentration. Inter-assay variability was 9.95% and within assay variability was 4.78%. Assays were performed blind. GDF15 level could not be measured in one participant leaving 1036 samples (331 dead and 705 alive) for the current analysis.

### Covariates

Baseline data were collected using a structured medical history interview and physical examination. Data collected included cigarette smoking, body mass index (BMI), waist circumference (WC), waist-hip ratio (WHR), diagnoses of hypertension and diabetes mellitus and estimated glomerular filtration rate [31]. Biochemical measures that were previously linked with mortality risk were assayed by Quest Diagnostics (Chantilly, VA). These variables were low density lipoprotein-cholesterol (LDL), high density lipoprotein-cholesterol (HDL), triglycerides, high-sensitivity C-reactive protein (hsCRP), red cell distribution width (RDW), mean corpuscular volume (MCV), albumin, albumin-globulin ratio (AGR), alkaline phosphatase (ALP) and blood urea nitrogen (BUN). LDL concentration was estimated based on total cholesterol, HDL and triglyceride levels using the Friedewald equation [32].

### Statistical analysis

Continuous variables were plotted using histograms, and skewness and excess kurtosis were calculated to assess their distribution. Skewed distribution was defined as skewness ≥ |1| and excess kurtosis ≥ 3. Variables with skewed distribution (GDF15, hsCRP, HDL, ALP and BUN) were natural-logarithm transformed to approximate a normal distribution. Categorical variables were sex, race (AAs and whites), poverty status (above and below the 125% federal poverty line), current cigarette smoking (yes, no), hypertension diagnosis (yes, no) and diabetes mellitus diagnosis (yes, no).

Unadjusted and multivariable adjusted Cox proportional hazards regression models were used to estimate the hazard ratios and 95% confidence intervals for all-cause mortality, CVD- and cancer-specific mortality for each unit increase in log-transformed GDF15 (logGDF15). Age at study entrance and exit (death or censored date December 31, 2016) in decimal years were used as the measurement of follow-up time [33]. The full Cox model was adjusted for sex, race, poverty status and an interaction term between race and poverty status. We included the interaction term between race and poverty status into the full Cox model because it was associated with all-cause mortality during initial model selection. We performed supplemental analysis by adding the following variables into the fully adjusted models: current cigarette smoking, hypertension diagnosis, diabetes mellitus diagnosis, BMI, WC, WHR, LDL, HDL, triglycerides, hsCRP, RDW, MCV, albumin, AGR, ALP, eGFR and BUN. These potentially confounding variables were added to the model to assess if they would substantially change the main findings of the paper.

We next assessed the interaction between logGDF15 and socioeconomic and demographic factors: sex, race and poverty status. Using forward model selection, we built Cox regression models for the three outcomes by including possible interaction terms between logGDF15 and sex, race and poverty status in the full Cox model described above. Significance of an interaction term between logGDF15 and any of the demographic variables was tested by comparing the models with and without the interaction term using a likelihood ratio test and significance value of 0.05.

The proportional hazards assumption for all unadjusted and multivariable adjusted Cox proportional hazards regression models were explored graphically with the use of *log (–log (survival))* plots and formally evaluated using the scaled Schoenfeld residuals chi-squared test resulting in global p values ≥ 0.34 indicating the proportional hazards assumption were not violated. We used calibration plots and the modified Nam-D’Agostino goodness-of-fit test to evaluate the contributions of logGDF15 and the interaction term containing logGDF15 to model performance for prediction of the three outcomes [34].

This study conforms to Strengthening the Reporting of Observational Studies in Epidemiology–Molecular Epidemiology (STROBE-ME) reporting guidelines for molecular epidemiology studies (www.equator-network.org). All data management and statistical data analyses were conducted with R v3.5.1 (www.r-project.org).

## Results

### Description of study participant characteristics

Description of the study participants are shown in Table 1. The age of study participants ranged from 30–64 years with mean age (standard deviation [SD]) of 49.6 (9.2). The proportion of women, AAs, above poverty status was 53.2%, 42.8% and 56.5%, respectively. During 12.2 years of follow-up (median = 9.2 years) with 8944.7 person-years, there were 331 deaths due to all-causes. The leading causes of death were CVD 94 (28.4%) followed by cancer 87 (26.3%). The median GDF15 level was 655.2 pg/mL (IQR = 575.1). Serum logGDF15 was moderately correlated with age (*r* = 0.45, p<0.001) and eGFR (*r* = -0.50, p<0.001) and weakly correlated with RDW (*r* = 0.21, p = <0.001) and BMI (*r* = -0.11, p<0.001) but not with hsCRP (*r* = 0.04, p = 0.20) or WC (*r* = -0.06, p = 0.09). Correlation coefficients between logGDF15 and other clinical and biochemical measures previously linked with organ-system functions and mortality risk are depicted in S1 Fig. The distribution of all-cause mortality events by race, poverty status and GDF15 quartiles is shown in S1 Table.

**Table 1:** Description of study participants with serum GDF15 and mortality data in the Healthy Aging in Neighborhoods of Diversity across the Life Span study.

Variables	Poverty status	
	Above	Below
	(n = 585)	(n = 451)
GDF15 (natural-log), mean (SD)	6.45 (0.65)	6.70 (0.73)
Age, mean (SD)	49.9 (9.2)	49.12 (9.1)
Sex, n (%)		
Women	299 (51.1)	252 (55.9)
Men	286 (48.9)	199 (44.1)
Race, n (%)		
Whites	281 (48.0)	162 (35.9)
African Americans	304 (52.0)	289 (64.1)
All-cause mortality, n	144	187
CVD-specific mortality, n	48	46
Cancer-specific mortality, n	40	47

Abbreviations: GDF15, growth differentiation factor 15; n, sample size; SD, standard deviation.

### Serum GDF15 and mortality outcomes

Unadjusted hazard ratio (HR) for all-cause mortality associated one unit of increase in logGDF15 was 2.41 (95% confidence interval [CI], 2.08–2.79). In the full Cox model adjusted for sex, race, poverty status and an interaction term between race×poverty status, the HR for all-cause mortality was 2.26 (95%CI, 1.94–2.64) (Table 2). The unadjusted and adjusted HR for CVD-specific mortality were 2.81 (95%CI, 2.13–3.70) and 2.74 (95%CI, 2.06–3.63), respectively. Similarly, logGDF15 was associated with cancer-specific mortality (all cancer types): unadjusted HR of 1.55 (95%CI, 1.11–2.16) and adjusted HR of 1.41 (95%CI, 1.00–2.00) (Table 2).

**Table 2:** Association between serum GDF15 and mortality risk in the Healthy Aging in Neighborhoods of Diversity across the Life Span study.

Model	N	All-cause mortality			CVD-specific mortality			Cancer-specific mortality		
		HR	95% CI		HR	95% CI		HR	95% CI	
Unadjusted model										
GDF15 (natural-log)	1036	2.41	2.08	2.79	2.81	2.13	3.7	1.55	1.11	2.16
Adjusted model*										
GDF15 (natural-log)	1036	2.26	1.94	2.64	2.74	2.06	3.63	1.41	1.00	2.00

* Model adjusted for sex, race, poverty status and race × poverty status interaction term.

Abbreviations: CI, confidence interval; CVD, cardiovascular diseases; GDF15, growth differentiation factor 15; HR, hazard ratio; N, sample size.

### Supplemental analysis

We performed supplemental analysis by further adjusting the full Cox model for the following variables: current cigarette smoking, hypertension diagnosis, diabetes mellitus diagnosis, BMI, WC, WHR, LDL, HDL, triglycerides, hsCRP, RDW, MCV, albumin, AGR, ALP, eGFR and BUN. The supplemental analyses did not materially change the association between logGDF15 and all-cause mortality risk and CVD-specific mortality risk (S2 Table). However, the association between logGDF15 and cancer-specific mortality become non-significant.

### Interaction between GDF15 and sex, race and poverty status

**Fig 1:**
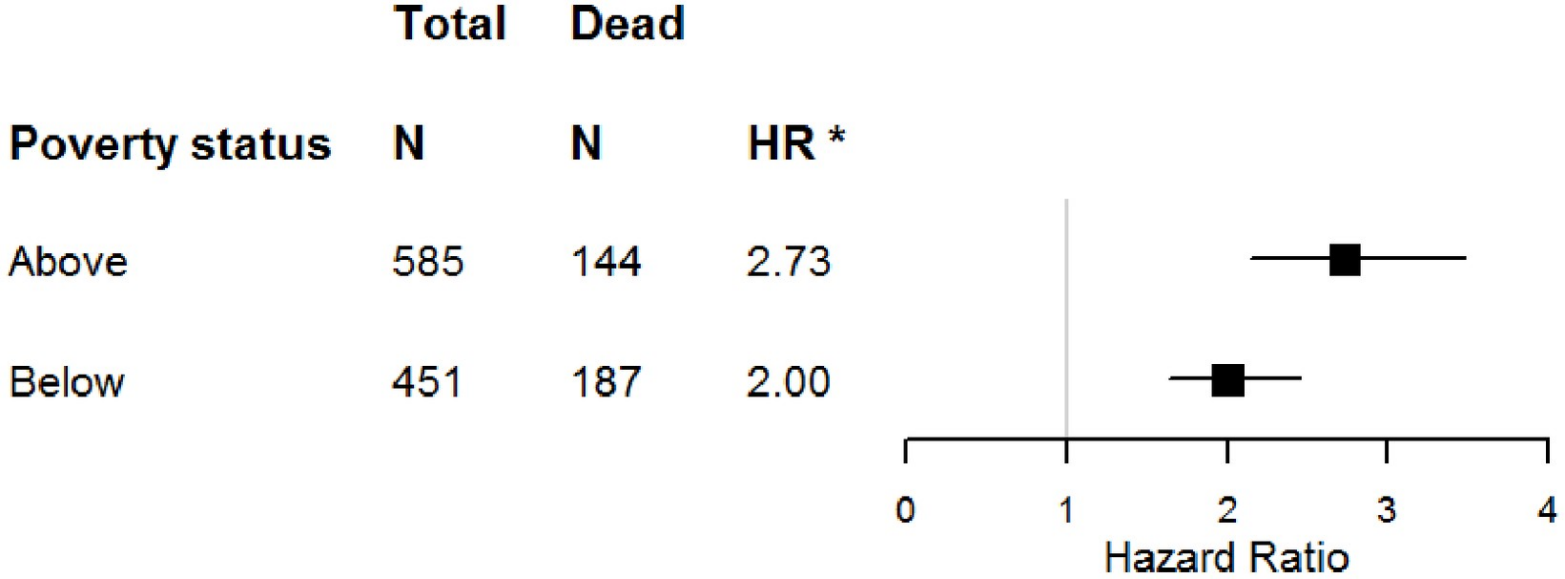
Association between serum GDF15 (natural-log) and all-cause mortality stratified by poverty status in the Healthy Aging in Neighborhoods of Diversity across the Life Span study. P value for interaction was 0.039. *Hazard ratio (HR) estimates were derived from the Cox proportional hazards regression models that controlled for sex and race. Abbreviations: HR, hazard ratio; N, sample size.

We assessed possible interactions between logGDF15 and sex, race and poverty status. There was a significant two-way interaction between logGDF15 and poverty status on all-cause mortality but not CVD- and cancer-specific mortality (Table 3). The increased risk of all-cause mortality associated with elevated logGDF15 was more pronounced in individuals above poverty level. In analyses that stratified participants by poverty status (above and below) and adjusted for sex and race, the HR for all-cause mortality in individuals above poverty was 2.73 (95%CI, 2.14–3.48) and for in individuals below poverty was 2.00 (95%CI, 1.64–2.45) (Fig 1). Model calibration test indicated logGDF15 and the interaction term logGDF15×poverty status improved outcome prediction of both all-cause and CVD-specific mortality (goodness-of-fit test p-values ≥ 0.42) (S2 Fig). We found no evidence of interaction between logGDF15 and sex or logGDF15 and race on any of the three outcomes.

**Table 3:** Interaction between serum GDF15 and poverty status on all-cause mortality risk in the Healthy Aging in Neighborhoods of Diversity across the Life Span study.

Variables	N	All-cause mortality		
		HR	95% CI	
GDF15 (natural-log)	1036	2.76	2.17	3.5
Sex				
Women	551	Reference		
Men	485	1.26	1.01	1.57
Race				
White	443	Reference		
African Americans	593	0.7	0.5	0.97
Poverty status				
Above	585	Reference		
Below	451	8.06	0.96	67.52
Interactions*				
Race × Poverty status	1036	2.19	1.38	3.48
GDF15 (natural-log) × Poverty status	1036	0.73	0.54	0.98

* Likelihood ratio test p value for interaction = 0.039. No interaction was found between serum GDF15 (natural-log) and poverty status on cardiovascular disease- and cancer-specific mortality risk.

Abbreviations: CI, confidence interval; GDF15, growth differentiation factor 15; HR, hazard ratio; N, sample size.

## Discussion

In a community-dwelling cohort of younger urban adults (mean age 49.6) with diverse racial and socioeconomic status, we found that elevated serum GDF15 level was strongly associated with all-cause mortality, CVD- and cancer-specific mortality risk. We also found an interaction between GDF15 and poverty status on all-cause mortality such that the association was more pronounced in individuals above poverty status than those below poverty status.

Our findings of increased risk of mortality due to all-causes and CVD and elevated GDF15 levels in a diverse cohort are consistent with results of previous studies conducted in apparently healthy, community-dwelling adults [12–15, 17, 18]. In three cohorts of community-dwelling elderly white men and women (mean age ranging from 68–78.6 years) from Sweden with baseline median GDF15 from 934.9 to 1393.0 pg/mL [13, 15], one unit increase in logGDF15 was associated with all-cause mortality with HRs ranging from 2.20 to 4.00 independent of traditional and established markers of mortality. Comparable risk estimates of CVD-specific mortality were also reported by these studies. In another Swedish male-only elderly cohort (mean age = 71.0 years) that had baseline median GDF15 of 1494.0 pg/mL, one SD increase in logGDF15 was associated with a 48% increase in all-cause mortality risk [14]. In our cohort of socioeconomically diverse AA and white men and women who were markedly younger than the Swedish cohort (mean age: 71.0 *versus* 49.6 years), one SD increase in logGDF15 conferred a 77% increased risk of all-cause mortality (95%CI, 1.59–1.97). In three population-based cohorts from the U.S.A., higher GDF15 levels were associated with increased risk of all-cause and CVD-specific mortality [12, 17, 18].

Our findings are also broadly comparable with other studies of GDF15 and mortality risk conducted in clinical trial participants, hospitalized patients and individuals with various chronic diseases. The comparability persists although these cohorts included highly select patient populations with advanced disease or critically ill patients who conceivably have high concentration of circulating GDF15 but are not representative of a general population like the HANDLS cohort [19, 35]. Collectively, these associations of GDF15 with mortality risk under diverse pathophysiological scenarios and study designs suggests that GDF15, a stress response cytokine, is a powerful biomarker that captures the body’s compensatory response reserve against persistent exposure to stressors and subsequent multi-organ and system failures due to various different diseases and co-morbidities, rather than just a reflection of a single disease burden or an isolated altered pathophysiological process.

We note that the median GDF15 concentration in the HANDLS cohort was significantly lower (655.2 pg/mL), almost half as much as previously reported in most studies of mortality risk in European-ancestry community-dwelling adults [12–15, 17]. However, in the Dallas Heart Study, comprised of multiethnic and younger participants, the median GDF15 value was 670 pg/mL, comparable to the GDF15 value in HANDLS [18]. This may suggest that even lower levels of GDF15 at a relatively younger age and many years before the onset of overt disease predict adverse health outcomes and could be a harbinger of mortality in the general population earlier in the lifespan. Given that midlife mortality rates (ages 25–64) have increased across racial/ethnic groups in the U.S.A. compounding the differential death rates already present among AAs, Native Americans and Alaska Natives, a useful biomarker of mortality risk among those at midlife could be a useful tool [3].

Our work provides the first evidence of interaction between GDF15 and poverty status on mortality risk. Compared to individuals below poverty, individuals above poverty had higher risk estimates (73%) of all-cause mortality. There are no previous studies that reported interactions between GDF15 and the social determinants of health on mortality risk in any context or study design. Previous population-based studies of mortality risk and GDF15 were conducted in largely homogenous population groups [12–15, 17] and neither measures of socioeconomic status (household income, education status, employment etc.) nor interactions were reported in these studies [12–15, 17, 18].

There is some evidence in animal models that GDF15 plays a role in lifespan. Two different transgenic mouse models that overexpress GDF15 showed increased lifespan, decreased adipose tissue weight, enhanced oxidative metabolism and improved insulin sensitivity, especially when challenged with high-fat diet [36]. Similarly, overexpression of GDF15 was shown to promote fatty acid oxidation and ketone body production and augmented the effects of prolonged fasting [23]. Therefore, it is plausible that GDF15 may reflect compensatory and adaptive response capacity and the ability to successfully mount an integrated organismal response to curb the effects of cellular and systemic stressors. It is possible that GDF15 is part of a cellular stress stimuli feedback loop that may protect from stress or inflammation and repair injury in selected biological settings while in other conditions promotes injury and disease [20].

Our observed interaction between GDF15 and poverty status on mortality risk is a bit counterintuitive. The expectation would be that mortality among those below poverty would be most detrimentally influenced by GDF15. This could be explained by differences between above and below poverty status individuals in adaptive response reserve and the ability to adequately respond to a multitude of stressors through increased GDF15 and possible additional molecular mechanisms. Indeed, several studies show that compared to high income individuals, those living in poverty and economic hardship across the life course, experience biological weathering evidenced by short leukocyte telomere length, the premature aging phenotype and accelerated biological aging [37–39]. This suggests that those living in poverty may be in a decompensated pathophysiological state lacking the homeostatic functional reserve necessary to respond to cellular stress and injury. This blunted ability to respond may preclude benefit from the protective effects of elevated GDF15 level. However, our finding that the influence of GDF15 on mortality is stronger among higher income individuals supports the previously described ‘diminishing returns hypothesis’ applied to various health outcomes among AAs including infant birthweight, depression, and self-rated health [40, 41]. The benefits of numerous socioeconomic resources are different for different races and perhaps the protective effects of these socioeconomic resources may also be related to neighborhood. In a recent study, race and living in an urban environment altered the protective effect of education on mortality among AAs [42]. Our finding is not a perfect fit since the central dogma of the diminishing returns hypothesis holds that AAs and, in some studies, Hispanics do not reap the expected health benefits of higher economic status perhaps related to the higher cost of coping with discrimination and race-based unequal treatment. In previous studies, whites have beneficial health outcomes from upward social mobility because of decrements in acute and chronic discrimination [43]. Our findings suggest that in our cohort diminishing returns holds true equally for younger middle-aged urban whites as well. In the context of the recently reported increasing mortality and declining life expectancy among middle-aged whites, it is possible that young middle-aged whites who are just slightly above the 125% poverty line may also be subject to diminishing returns from the protective benefits of socioeconomic resources and that this correlates with the observed increases in mortality [3]. This could result from changes in economic or social factors that influence health outcomes for whites who are at the lower end of the income scale when compared to other whites in the Baltimore City.

Within race income inequality has increased substantially nationwide among all U.S. racial groups. While it is highest among U.S. Asians, since 1970 income inequality among whites has risen by 24%; in 2016 whites at the 90^th^ percentile income distribution made 7.8 times the income of whites at the 10^th^ percentile income distribution [44]. Baltimore City has the highest income inequality gap in Maryland and this may not only influence health outcomes among AAs but also whites [45]. In our cohort, two-thirds of those living below poverty status had an annual household income below $30 000. In Baltimore the median household income is $44 262: for whites $62 752 and for AAs $33 801 [46]. In HANDLS, those living above poverty status were more affluent, but only 25% had an annual household income over $50 000.

Future studies are required to understand the exact biology behind the interrelationship between GDF15 and stressful life circumstances, as in living in poverty, on adverse health outcomes. It is also important to understand how temporal changes in GDF15 levels vis-à-vis socioeconomic measures relate with mortality risk and aging-related diseases in disadvantaged communities and to assess interaction between changes in GDF15 and the social determinants of health. In a study of elderly individuals of European-ancestry, serum GDF15 levels increased by 11% after five years of follow-up and this rate of change was associated with overall survival [13]. This study highlights the importance of conducting a longitudinal assessment in middle-aged individuals to assess rate of change and survival.

The limitations of our study are (1) smaller number of mortality events among whites below poverty status precluded three-way interaction analysis between race, poverty and GDF15; (2) similarly smaller number of individuals with specific causes of death limited further disease-specific survival analysis; (3) other important factors such as mental health diagnoses that might be a confounding variable influencing poverty, GDF15, and mortality were not assessed.

Our study has several strengths including large sample size, contemporary cohort that captures recent mortality trends and their cause and is comprised of younger, socioeconomically and racially diverse adults. Previous GDF15 and mortality studies were conducted in predominantly elderly men and of homogenous European-ancestry populations [12–15, 17]; of note here is that 4 out of the 7 community-dwelling cohorts were from a single Scandinavian country [13–15]. While we used poverty thresholds, which is based on annual household income used by the U.S. federal government, application of a more nuanced measures of poverty and socioeconomic stress when evaluating the relationship between GDF15, socioeconomic hardship and mortality risk could enhance understanding.

In conclusion, elevated GDF15 levels are associated with increased risk of mortality, and that this association was modified by poverty status. This is the first time that a social determinant of health has been shown to enhance the risk of a biomarker of mortality. While GDF15 has independent prognostic value as a single mortality marker, the true value of GDF15 might be enhanced by coupling it with other routinely obtained clinical markers of disease and mortality including hs-CRP, RDW and the social determinant of health poverty as well as assessment of mental health status to more accurately define those at highest risk for negative health outcomes. Future studies are needed to extend our findings in other population groups. Further studies are also required to explore the potential of targeting GDF15 to improve mortality and other health outcomes.

## Supporting information

S1 Fig Correlation between serum GDF15 and clinical and biochemical measures in HANDLS. (DOCX)

S2 Fig Evaluation of model calibration using the modified Nam-D’Agostino goodness-of-fit test with observed and expected mortality counts in HANDLS. (DOCX)

S1 Table P values of association between log GDF15 and mortality events following further adjustment for clinical and biochemical measures linked with mortality risk in prior studies. (DOCX)

S2 Table Distribution of all-cause mortality status by race and poverty status in HANDLS. (DOCX)
