# Supplementary material for: Association between GDF15, poverty and mortality in urban middle-aged African American and white adults
Source: PLoS One. 2020 Aug 7;15(8):e0237059. doi: 10.1371/journal.pone.0237059 (PMC7413478; doi:10.1371/journal.pone.0237059)
Supplement: S1 Fig — (DOCX) [file pone.0237059.s001.docx]

**Freeman *et al.*, Association between GDF15, poverty and mortality in urban middle-aged African American and white adults**


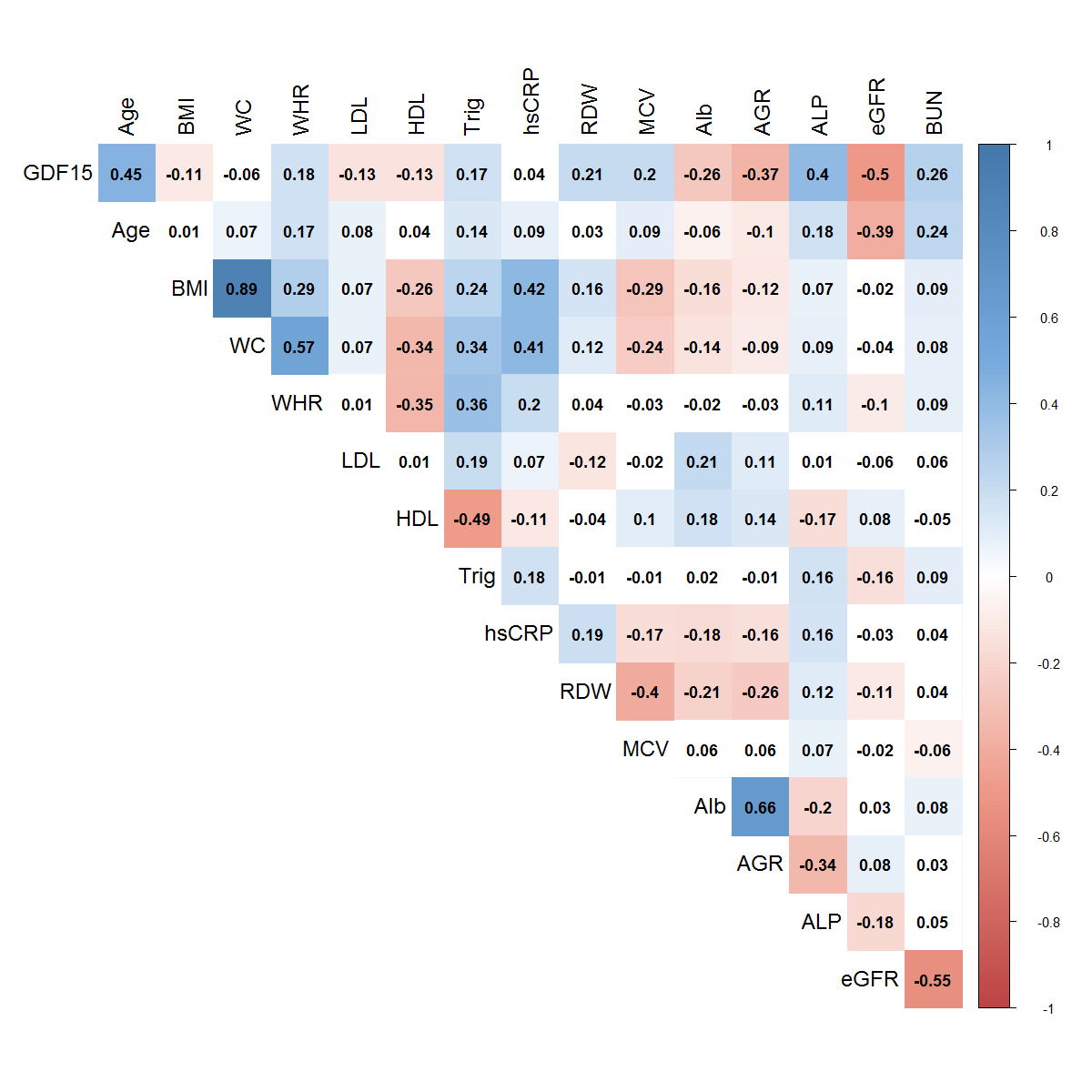


**S1 Fig.** Correlation between serum GDF15 and clinical and biochemical measures in the Healthy Aging in Neighborhoods of Diversity across the Life Span study (2004 – 2016). Correlation coefficients with p values < 0.05 are highlighted in blue (positive correlation) or red (negative correlation).

Abbreviations: Alb, albumin; AGR, albumin-globulin ratio; ALP, alkaline phosphatase; BMI, body mass index; BUN, blood urea nitrogen; hsCRP, high sensitivity C-reactive protein; eGFR, estimated glomerular filtration rate; GDF15, growth differentiation factor 15; HDL, high density lipoprotein-cholesterol; LDL, low density lipoprotein-cholesterol; MCV, mean corpuscular volume; RDW, red cell distribution width; Trig, triglycerides; WC, waist circumference; WHR, waist-hip ratio.
