# Supplementary material for: Association between GDF15, poverty and mortality in urban middle-aged African American and white adults
Source: PLoS One. 2020 Aug 7;15(8):e0237059. doi: 10.1371/journal.pone.0237059 (PMC7413478; doi:10.1371/journal.pone.0237059)
Supplement: S2 Fig — (DOCX) [file pone.0237059.s002.docx]

(**a**) (**b**)
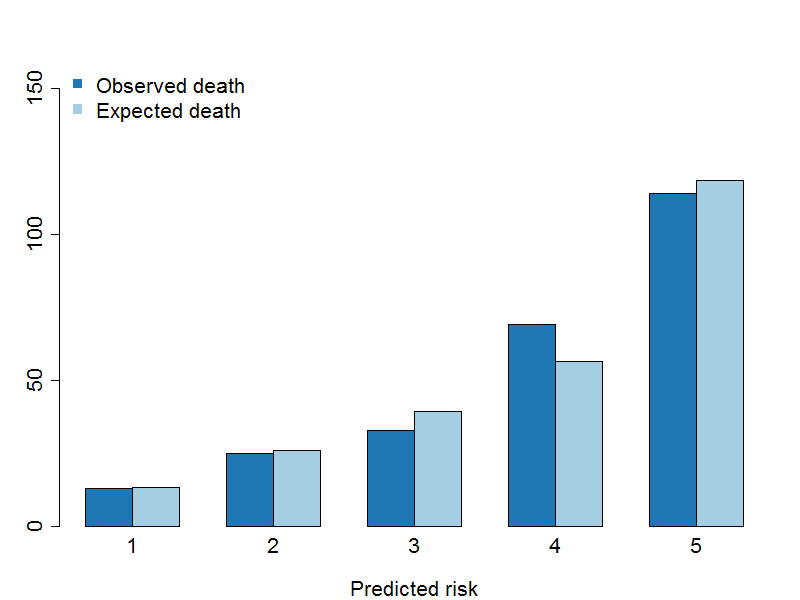

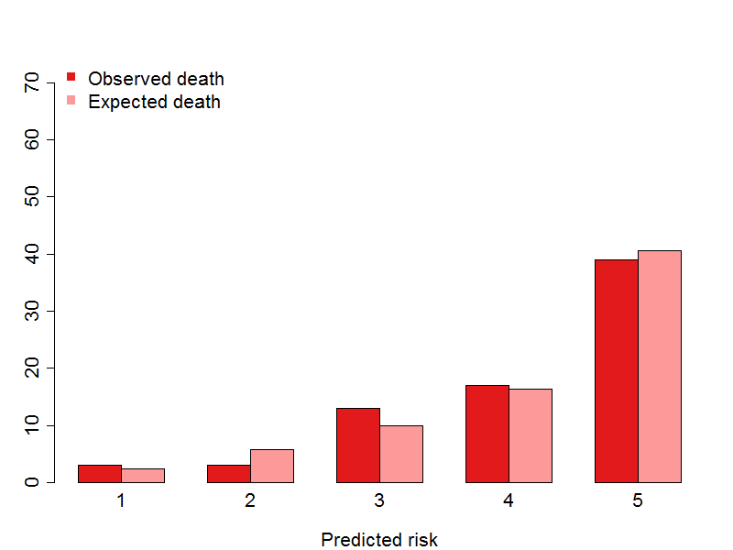


**S2 Fig.** Evaluation of model calibration using the modified Nam-D’Agostino goodness-of-fit test with observed and expected mortality counts in the Healthy Aging in the Neighborhoods of Diversity across the Life Span study (2004 – 2016). Mortality counts were grouped into five bins. The addition of (**a**) both natural-log transformed GDF15 (logGDF15) and the interaction term logGDF15 × poverty status into the Cox proportional hazards regression model of all-cause mortality [χ^2^ = 3.92, df = 4, p = 0.42], and (**b**) logGDF15 into the Cox proportional hazards regression models of CVD-specific mortality improved model performance [χ^2^ = 0.82, df = 4, p = 0.94].
