# Supplementary material for: Association between GDF15, poverty and mortality in urban middle-aged African American and white adults
Source: PLoS One. 2020 Aug 7;15(8):e0237059. doi: 10.1371/journal.pone.0237059 (PMC7413478; doi:10.1371/journal.pone.0237059)
Supplement: S1 Table — (DOCX) [file pone.0237059.s003.docx]

**S1 Table.** Distribution of all-cause mortality status by race and poverty status in the Healthy Aging in the Neighborhoods of Diversity across the Life Span study (2004 – 2016).

| Race | Poverty status | GDF15 (natural-log) | All-cause mortality status | |
| --- | --- | --- | --- | --- |
|  |  |  | Alive, n = 705 | Dead, n = 331 |
|  |  |  |  |  |
| Whites | Above | Quartile1 | 79 | 10 |
|  |  | Quartile2 | 62 | 14 |
|  |  | Quartile3 | 44 | 23 |
|  |  | Quartile4 | 21 | 28 |
|  | Below | Quartile1 | 20 | 3 |
|  |  | Quartile2 | 34 | 4 |
|  |  | Quartile3 | 36 | 9 |
|  |  | Quartile4 | 20 | 36 |
| African Americans | Above | Quartile1 | 80 | 4 |
|  |  | Quartile2 | 69 | 13 |
|  |  | Quartile3 | 55 | 20 |
|  |  | Quartile4 | 31 | 32 |
|  | Below | Quartile1 | 49 | 14 |
|  |  | Quartile2 | 42 | 21 |
|  |  | Quartile3 | 37 | 35 |
|  |  | Quartile4 | 26 | 65 |
| Abbreviation: GDF15, growth differentiation factor 15. | | | | |
