# Supplementary material for: Association between GDF15, poverty and mortality in urban middle-aged African American and white adults
Source: PLoS One. 2020 Aug 7;15(8):e0237059. doi: 10.1371/journal.pone.0237059 (PMC7413478; doi:10.1371/journal.pone.0237059)
Supplement: S2 Table — (DOCX) [file pone.0237059.s004.docx]

**S2 Table.** P values of association between log GDF15 and mortality events following further adjustment for clinical and biochemical measures linked with mortality risk in prior studies: Healthy Aging in Neighborhoods of Diversity across the Life Span study (2004 – 2016).

| Variables | N _total_ | **All-cause mortality** | | **CVD-specific mortality** | | **Cancer-specific mortality** | |
| --- | --- | --- | --- | --- | --- | --- | --- |
|  |  | N _event_ | P _GDF15_ | N _event_ | P _GDF15_ | N _event_ | P _GDF15_ |
| Current cigarette smoking | 944 | 300 | 2.99E-19 | 87 | 3.98E-10 | 76 | 4.28E-01 |
| Hypertension | 965 | 312 | 2.09E-21 | 89 | 2.07E-09 | 81 | 2.87E-02 |
| Diabetes mellitus | 983 | 316 | 4.03E-21 | 87 | 1.89E-08 | 83 | 2.42E-02 |
| Body mass index | 1006 | 320 | 1.49E-23 | 91 | 8.49E-12 | 83 | 2.07E-01 |
| Waist circumference | 970 | 304 | 1.46E-20 | 85 | 2.04E-10 | 80 | 2.28E-01 |
| Waist-hip ratio | 970 | 304 | 1.45E-20 | 85 | 2.36E-09 | 80 | 1.14E-01 |
| LDL | 947 | 289 | 1.35E-21 | 80 | 1.92E-10 | 78 | 7.36E-02 |
| HDL (natural-log) | 1033 | 331 | 3.40E-25 | 94 | 9.19E-12 | 87 | 3.50E-02 |
| Triglyceride | 967 | 299 | 2.86E-24 | 85 | 4.25E-10 | 79 | 3.30E-02 |
| hsCRP (natural-log) | 1036 | 331 | 3.70E-25 | 94 | 9.91E-12 | 87 | 5.14E-02 |
| RDW | 1024 | 326 | 4.24E-23 | 92 | 4.03E-10 | 86 | 4.84E-02 |
| MCV | 1030 | 329 | 7.63E-24 | 94 | 1.86E-12 | 86 | 1.07E-01 |
| Albumin | 1027 | 325 | 6.38E-19 | 92 | 4.60E-11 | 87 | 4.54E-02 |
| AGR | 1033 | 331 | 5.76E-16 | 94 | 1.37E-09 | 87 | 1.60E-01 |
| ALP (natural-log) | 1032 | 330 | 6.16E-16 | 93 | 1.82E-07 | 87 | 1.28E-01 |
| eGFR | 994 | 322 | 2.49E-25 | 92 | 4.83E-07 | 84 | 9.02E-03 |
| BUN (natural-log) | 1029 | 327 | 6.18E-26 | 92 | 2.67E-10 | 86 | 3.17E-02 |
| Abbreviations: AGR, albumin-globulin ratio; ALP, alkaline phosphatase; BUN, blood urea nitrogen; CVD, cardiovascular disease; hsCRP, high-sensitivity C-reactive protein; eGFR, estimated glomerular filtration rate; GDF15, growth differentiation factor 15; HDL, high density lipoprotein-cholesterol; LDL, low density lipoprotein-cholesterol; MCV, mean corpuscular volume; N, sample size; RDW, red cell distribution width. | | | | | | | |
